# Hepatocyte Autophagy in Malaria: Current Concepts, Emerging Mechanisms, and Future Therapeutic Directions

**DOI:** 10.3390/pathogens15010070

**Published:** 2026-01-09

**Authors:** Afiat Berbudi, Shafia Khairani, Endang Yuni Setyowati, Alexander Kwarteng

**Affiliations:** 1 Department of Biomedical Sciences, Parasitology Division, Faculty of Medicine, Universitas Padjadjaran, Bandung 40161, Indonesia; 2 Research Center for Care and Control of Infectious Diseases (RC3ID), Universitas Padjadjaran, Bandung 40161, Indonesia; 3 Department of Biomedical Sciences, Cell Biology Division, Faculty of Medicine, Universitas Padjadjaran, Bandung 40161, Indonesia; shafia@unpad.ac.id; 4 Veterinary Medicine Program, Faculty of Medicine, Universitas Padjadjaran, Sumedang 45363, Indonesia; endang.yuni@unpad.ac.id; 5 Department of Biochemistry and Biotechnology, Kwame Nkrumah University of Science and Technology, PMB University Post Office, Kumasi, Ghana; akwarteng@knust.edu.gh

**Keywords:** autophagy, liver, malaria, *Plasmodium*, host–pathogen interactions, endosomes

## Abstract

The liver stage of *Plasmodium* infection represents a critical bottleneck in malaria pathogenesis and a unique interface between parasite development and hepatocyte-intrinsic immunity. Recent evidence suggests that hepatocytes do not eliminate liver-stage parasites through canonical xenophagy, as previously assumed, but instead employ a noncanonical autophagy response known as the conjugation of ATG8 to single membranes (CASM). CASM drives rapid lipidation of LC3 onto the parasitophorous vacuole membrane (PVM) via a V-ATPase-ATG16L1-dependent mechanism, thereby activating the *Plasmodium*-associated autophagy-related (PAAR) response. This process represents a major hepatocyte-intrinsic mechanism that limits early liver-stage parasite development. *Plasmodium* liver-stage parasites have evolved specialized strategies to counteract this host defense. The PVM proteins UIS3 and UIS4 enable parasite evasion by sequestering LC3 and remodeling perivacuolar actin, thereby preventing endolysosomal fusion and inhibiting PAAR execution. In parallel, parasites selectively exploit host autophagy components—particularly GABARAP paralogs—to activate TFEB, promoting lysosomal biogenesis and improving access to host-derived nutrients. These interactions highlight autophagy as both a protective and parasite-supportive pathway, depending on the molecular context. Understanding how CASM, PAAR, and parasite evasion mechanisms intersect is crucial for designing pathway-selective interventions that amplify hepatocyte-intrinsic clearance while avoiding the inadvertent enhancement of parasite-supportive autophagy programs. Selective modulation of noncanonical autophagy offers a promising avenue for host-directed therapies that restrict liver-stage development while limiting the emergence of antimalarial resistance. This review synthesizes recent advances in the mechanistic interplay between *Plasmodium* liver stages and hepatocyte autophagy, identifies major knowledge gaps, and outlines future directions for translating these discoveries into therapeutic innovation.

## 1. Introduction

Malaria remains a major global health challenge, with over 263 million cases reported annually and continued transmission despite widespread deployment of vector control, chemoprevention, and artemisinin-based combination therapies [1]. The pre-erythrocytic liver stage represents a critical bottleneck in the *Plasmodium* life cycle, during which a small number of sporozoites infect hepatocytes and undergo extensive replication before initiating symptomatic blood-stage infection [2,3]. Because parasite biomass is low and genetically non-diverse at this stage, the liver stage offers an attractive target for both prophylactic vaccines and host-directed therapies aimed at preventing progression to clinical disease [4,5].

Once inside hepatocytes, *Plasmodium* develops within a modified parasitophorous vacuole membrane (PVM), which acts as the primary interface for host–pathogen interactions [6,7,8]. Host cells mount a rapid cytosolic defense response upon parasite invasion, including interferon-driven immune pathways and endolysosomal remodeling [9,10,11]. A key component of this response is the recruitment of LC3 (microtubule-associated protein 1 light chain 3), a member of the ATG8 (autophagy-related protein 8) family, to the PVM [7,12,13]; however, studies primarily using *Plasmodium berghei* liver-stage infection models have demonstrated that this process does not reflect classical xenophagy but instead represents a form of noncanonical autophagy [13,14,15].

This pathway, known as conjugation of ATG8 to single membranes (CASM), a form of noncanonical autophagy distinct from classical double-membrane autophagosome formation, is initiated by V-ATPase-dependent recruitment of ATG16L1 and leads to LC3 lipidation on the single-membrane PVM, forming the *Plasmodium*-associated autophagy-related (PAAR) response [15]. Most mechanistic insights into CASM/PAAR during malaria have been derived from liver-stage experimental models, and the degree of conservation across *Plasmodium* species and clinical malaria settings remains to be fully established. PAAR represents an early cell-intrinsic defense that limits parasite survival and developmental progression within hepatocytes—a process referred to here as parasite restriction, defined as the suppression of parasite growth, maturation, or persistence through host-mediated intracellular mechanisms rather than direct parasite killing [16,17].

To counteract this response, *Plasmodium* expresses PVM-resident proteins such as UIS3 and UIS4, which interfere with host autophagy by sequestering LC3 through direct binding and functional immobilization at the parasitophorous vacuole membrane and by driving perivacuolar actin remodeling, thereby limiting lysosomal access [12,18,19]. At later stages, parasites exploit host autophagy machinery by selectively recruiting GABARAP (γ-aminobutyric acid receptor-associated protein) paralogs, a subset of ATG8-family proteins involved in vesicular trafficking and autophagy regulation, and activating TFEB (transcription factor EB), a master regulator of lysosomal biogenesis, thereby enhancing access to lipids and nutrients required for intracellular development [20,21,22,23].

Together, these findings reveal that autophagy plays a dual role in liver-stage malaria—both restricting and supporting parasite development depending on the molecular context. Importantly, these interactions reveal an inherently asymmetric landscape in which hepatocyte-intrinsic restriction and parasite-driven exploitation can operate in parallel, often drawing on overlapping autophagy machinery but yielding opposite outcomes. A comprehensive understanding of CASM activation, parasite evasion mechanisms, and autophagy-mediated metabolic remodeling—defined here as parasite-driven reprogramming of hepatocyte lysosomal biogenesis, membrane trafficking, and lipid mobilization through selective engagement of autophagy signaling pathways—is therefore crucial for designing selective, host-directed strategies to prevent liver-stage progression and reduce the emergence of antimalarial resistance. This review synthesizes current advances in the mechanistic interplay between *Plasmodium* liver stages and hepatocyte autophagy. Unless otherwise specified, the majority of mechanistic insights discussed here are derived from studies using the rodent malaria parasite *Plasmodium berghei*, which remains the dominant experimental model for liver-stage autophagy research. Where available, evidence from *Plasmodium falciparum* or human-relevant systems is explicitly indicated.

## 2. Mechanistic Architecture of Autophagy During *Plasmodium* Liver-Stage Infection

The liver stage of *Plasmodium* infection begins with sporozoite entry into hepatocytes, a process traditionally viewed as parasite-driven but now understood to involve substantial host contributions. Live-cell imaging studies demonstrate that the initial contact between motile sporozoites and hepatocytes induces pronounced plasma membrane ruffling and filopodia extension, driven in part by Rho GTPase signaling, which increases host cell susceptibility to productive invasion [24]. These host-directed membrane dynamics facilitate plasma membrane invagination, giving rise to the single-membrane Parasitophorous vacuole membrane (PVM) that envelops the intracellular parasite and provides the structural platform for subsequent liver-stage development [25,26]. Once formed, the PVM becomes the central interface for host–parasite interaction and a focal point for a specialized form of noncanonical autophagy, CASM. Recent work has shown that CASM—rather than canonical xenophagy—governs LC3 recruitment to the PVM through V-ATPase–ATG16L1-dependent lipidation, constituting a major hepatocyte-intrinsic defense mechanism during early liver-stage infection [14,15].

### 2.1. Activation of Noncanonical Autophagy (CASM) at the PVM

Upon productive invasion, the PVM undergoes rapid biophysical and ionic perturbations—including changes in membrane tension, lipid organization, and local ion fluxes such as proton and calcium gradients—that serve as upstream signals for the recruitment of the V-ATPase complex and the ATG16L1–ATG5–ATG12 machinery to the vacuole surface [27,28]. Unlike canonical autophagy, which is a degradative pathway characterized by the formation of double-membrane autophagosomes initiated by the ULK1 and Beclin1–VPS34 complexes, these initiation pathways instead enable the direct conjugation of LC3 to the single-membrane PVM bilayer [29,30].

**Figure 1:**
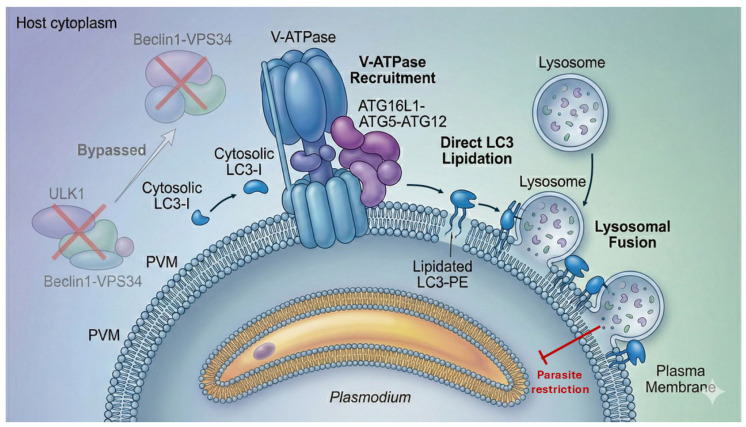
Schematic overview of noncanonical autophagy targeting the *Plasmodium* parasitophorous vacuole membrane (PVM) in hepatocytes. Upon parasite invasion, host cells initiate a defense response in which LC3 is directly attached to the single-membrane PVM, rather than forming a classical double-membrane autophagosome. This process occurs independently of canonical autophagy initiation complexes, such as ULK1 and Beclin1–VPS34 (indicated by red “X” symbols). Recruitment of the V-ATPase and ATG16L1–ATG5–ATG12 complex enables LC3 lipidation on the PVM, leading to lysosomal fusion and restriction of parasite development (red arrow). Directional arrows indicate the recruitment and trafficking of host autophagy components toward the PVM.

This modification defines the PAAR—a hallmark of hepatocyte defense—characterized by long-lasting LC3B decoration of the PVM [15,27]. LC3 lipidation marks the PVM for interaction with endolysosomal machinery, enabling downstream tethering events required for parasite restriction (Figure 1).

### 2.2. LC3-Dependent Restriction and Lysosomal Engagement

LC3-decorated PVMs can recruit ubiquitin and autophagy adaptors, enhancing the recognition of the Parasitophorous compartment as damaged or foreign [9,12]. Subsequent fusion with host lysosomes leads to parasite degradation through a nitric oxide-independent mechanism, redefining the centrality of noncanonical autophagy in parasite elimination [10,16]. These observations collectively establish that CASM-driven LC3 lipidation, not classical xenophagy, is the predominant autophagy pathway engaged by hepatocytes during early liver-stage infection. Disruption of V-ATPase or ATG16L1 significantly impairs LC3 recruitment and compromises parasite clearance, confirming CASM as a core arm of hepatocyte cell-autonomous immunity [15].

### 2.3. Parasite Interference with CASM Execution

To survive within the hepatocyte, *Plasmodium* deploys specialized PVM-resident proteins that interfere with the execution—but not necessarily the initiation—of the PAAR response. The best-characterized factor, UIS3, identified primarily in *Plasmodium berghei*, binds directly to LC3B, acting as a molecular sink that prevents LC3-engaged PVMs from progressing toward lysosomal fusion [12,31]. In parallel, UIS4, characterized in rodent malaria models, organizes actin cytoskeleton remodeling around the PVM, creating a dense perivacuolar barrier that restricts access of lysosomes, ubiquitin, and autophagy adaptors [18,19]. These evasion mechanisms do not abolish LC3 lipidation but instead derail the maturation of CASM into a fully restrictive pathway, allowing the parasite to remain protected despite early detection by hepatocyte surveillance systems. It is important to note, however, that the molecular mechanisms underlying UIS3-mediated LC3 sequestration have been primarily delineated using in vitro hepatocyte models, including transformed hepatoma cell lines and primary hepatocytes, often under highly synchronized infection conditions [12]. While complementary rodent in vivo studies, such as UIS3 depletion models, confirm the essential role of UIS3 for liver-stage development [31], these systems do not fully recapitulate the metabolic heterogeneity, cellular architecture, and immune complexity of the human liver. Consequently, the quantitative contribution and regulatory dynamics of UIS3-dependent autophagy evasion in human *Plasmodium* infections remain to be fully resolved.

### 2.4. Divergent ATG8 Paralog Engagement

While LC3B is associated primarily with parasite restriction, liver-stage parasites selectively recruit GABARAP paralogs at later developmental stages [32]. Recent work shows that GABARAP enrichment at the PVM promotes TFEB activation, the master regulator of lysosomal biogenesis and autophagy gene expression [20]. Unlike LC3-driven PAAR, this pathway enhances intracellular trafficking capacity and lipid mobilization—processes that support parasite replication and metabolic expansion [21]. This differential ATG8 engagement underscores a fundamental asymmetry in the autophagy landscape:

- LC3 → Restrictive, CASM-dependent defense;
- GABARAP → Supportive, TFEB-dependent exploitation.

Understanding the determinants of this paralog specificity remains a key unresolved question.

Together, these mechanistic insights define the molecular architecture of noncanonical autophagy at the *Plasmodium* PVM. CASM-driven LC3 lipidation, lysosomal tethering, and the counteracting roles of UIS3 and UIS4, along with selective ATG8 recruitment, illustrate that the hepatocyte–parasite interface is shaped by tightly regulated, molecule-specific interactions rather than a uniform autophagic program. However, these mechanisms do not operate in isolation. Instead, they form a dynamic and competitive landscape in which hepatocyte-intrinsic defense and parasite-driven exploitation unfold simultaneously and often asymmetrically. To understand how these discrete molecular events translate into infection outcomes, it is necessary to move beyond individual pathways and examine how host and parasite autophagy programs intersect, oppose, or reinforce each other. Section 3 synthesizes these interactions into an integrated conceptual framework that captures the emergent behavior of the autophagy interface during liver-stage malaria.

## 3. Integrated Host–Parasite Autophagy Dynamics During Liver-Stage Malaria

The molecular events described in Section 2 reveal that hepatocytes initiate a CASM-driven PAAR response that decorates the PVM with LC3 and promotes lysosomal engagement. However, the liver stage of *Plasmodium* infection is not defined solely by isolated autophagy pathways. Instead, it reflects a dynamic and asymmetric interplay in which hepatocyte restriction mechanisms and parasite survival strategies operate simultaneously, often competing for the same autophagy machinery. This section synthesizes these interactions into a unified conceptual framework that captures how noncanonical autophagy shapes liver-stage infection outcomes.

### 3.1. Host PAAR/CASM as a Restrictive Autophagy Pathway

Hepatocytes deploy PAAR as a form of cell-autonomous immunity, characterized by persistent LC3 lipidation onto the single-membrane PVM through V-ATPase-ATG16L1-dependent CASM activation [14,15]. This LC3 enrichment enables the PVM to be recognized by ubiquitin and autophagy adaptors, allowing it to be tethered to lysosomes, which culminates in parasite degradation independent of nitric oxide [10,16]. At the systems level, PAAR represents an early restriction axis that acts during the narrow temporal window immediately following sporozoite invasion, when parasite numbers are low and host defense is at its peak.

To consolidate the mechanistic insights outlined above, we provide a structured summary of the key components involved in hepatocyte CASM/PAAR activation. Table 1 synthesizes the major molecular triggers, autophagy machinery, and downstream restrictive processes that shape the early host response to *Plasmodium* liver-stage infection. This table provides a concise reference to the core mechanisms underlying noncanonical autophagy at the PVM.

### 3.2. Parasite Evasion of PAAR Execution

**Figure 2:**
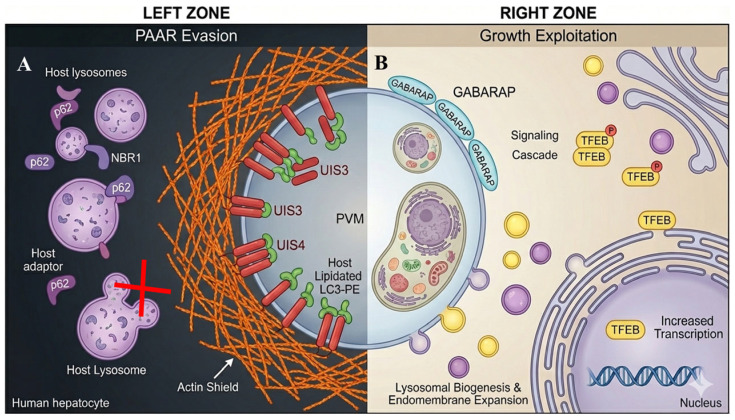
Divergent mechanisms at the *Plasmodium* PVM interface: evasion vs. exploitation. Schematic illustration of a hepatocyte cross-section showing an elongated *Plasmodium* parasite enclosed within the PVM. The interface is conceptualized into two functional zones: (**A**) Left (PAAR evasion) depicts parasite defense strategies. The parasite transmembrane protein UIS3 binds and sequesters host LC3 on the PVM. Concurrently, UIS4 induces a dense, chaotic actin filament shield, physically preventing host lysosomes and autophagy adaptors from fusing with the PVM. (**B**) Right (growth exploitation) shows metabolic hijacking. The PVM selectively recruits the host ATG8 paralog, GABARAP, initiating a signaling cascade that phosphorylates and activates the transcription factor TFEB. Activated TFEB translocates to the host nucleus, upregulating lysosomal biogenesis and endomembrane expansion, which are subsequently diverted to the parasite for nutrient supply. Colors and symbols are used for clarity: orange filaments indicate the actin-based shield, purple vesicles represent host lysosomes, green labels denote LC3/GABARAP-associated components, yellow circles indicate TFEB-related signaling, and arrows show the direction of host–parasite interactions at the PVM.

To withstand PAAR-mediated restriction, *Plasmodium* deploys targeted interference strategies that divert or block the downstream maturation of LC3-decorated PVMs. UIS3 binds LC3 directly, preventing its recruitment of degradative adaptors and effectively trapping LC3 in a non-restrictive configuration [12,31]. In parallel, UIS4 remodels the perivacuolar actin network to create a dense physical barrier that limits access of lysosomes and ubiquitin (Figure 2) [18,19]. These mechanisms do not inhibit CASM initiation; instead, they selectively target the execution phase of PAAR. As a result, hepatocytes continue to recognize and label the PVM with LC3, but parasites prevent the transition from LC3 decoration to effective lysosomal killing. This creates a biological stalemate in which LC3 recruitment becomes necessary but insufficient for eliminating the parasite.

### 3.3. Parasite Exploitation of Autophagy Components for Intracellular Growth

Beyond PAAR evasion, liver-stage parasites co-opt host autophagy machinery to support their metabolic expansion. Recent work using *Plasmodium berghei* liver-stage parasites shows selective recruitment of GABARAP paralogs—distinct members of the ATG8 family with trafficking and signaling functions—to the PVM at later stages [20,32]. Unlike LC3, which drives restriction, GABARAP promotes activation of TFEB, the master transcriptional regulator of lysosomal biogenesis and autophagy gene expression (Figure 2) [23].

TFEB activation expands the hepatocyte’s lysosomal network and increases endomembrane trafficking capacity, generating a nutrient-rich and membrane-rich environment that accelerates parasite replication [20,21]. Through this strategy, the parasite effectively repurposes host autophagy infrastructure to meet its metabolic and organellar demands during schizogony.

Given that hepatocyte CASM/PAAR activity is counterbalanced by parasite-driven evasion and exploitation mechanisms, it is essential to contextualize these opposing processes within a unified framework. Table 2 summarizes the principal strategies *Plasmodium* uses to interfere with LC3-mediated restriction and to co-opt autophagy components for selective metabolic advantage. This overview highlights how molecular evasion (UIS3 and UIS4) and metabolic exploitation (GABARAP-TFEB signaling) collectively reshape the autophagy landscape at the PVM.

### 3.4. An Asymmetric Autophagy Landscape: Restriction vs. Exploitation

**Figure 3:**
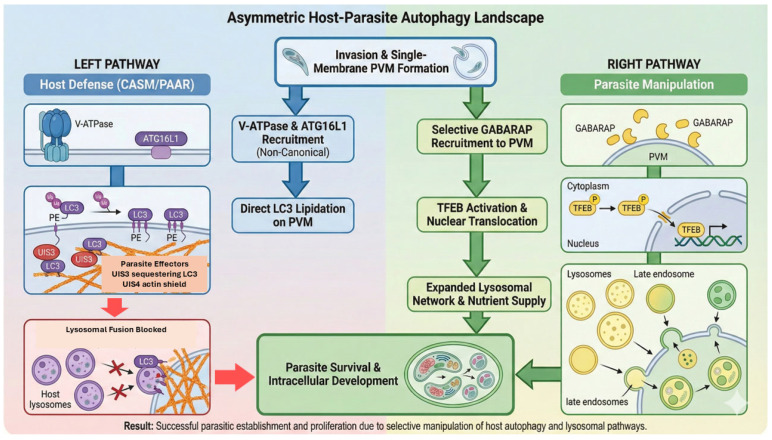
The asymmetric host–parasite autophagy landscape: defense vs. exploitation. This schematic summarizes the divergent autophagy pathways occurring at the PVM interface. (**Left**) Host defense: the host initiates cell-autonomous immunity (CASM/PAAR) by recruiting V-ATPase and ATG16L1 to facilitate direct LC3 lipidation on the PVM, thereby driving lysosomal fusion. However, this restriction mechanism is counteracted by parasite effectors (UIS3 sequestration and the UIS4-mediated actin shield), which effectively block lysosomal attack. (**Right**) Parasite manipulation: concurrently, the parasite exploits the pathway by selectively recruiting the host ATG8 paralog, GABARAP. This recruitment triggers a signaling cascade leading to the activation and nuclear translocation of TFEB. This results in upregulated lysosomal biogenesis and endomembrane expansion, providing an expanded nutrient supply essential for parasite survival and development. Colors and symbols are used for clarity, and arrows indicate the direction of host–parasite interactions and pathway progression at the PVM.

Taken together, the host and parasite simultaneously engage autophagy pathways in a fundamentally asymmetric manner. LC3-driven CASM/PAAR represents a restrictive pathway that hepatocytes deploy to eliminate intracellular parasites. GABARAP-dependent TFEB activation, by contrast, represents an exploitative pathway hijacked by the parasite to enhance intracellular growth. These pathways use overlapping autophagy components but yield opposite biological outcomes. This asymmetry yields three key conceptual insights (Figure 3):

- Autophagy is not uniformly protective: its impact depends on which ATG8 paralog predominates at the PVM.
- Host defense and parasite survival occur in parallel: evasion mechanisms (UIS3 and UIS4) prevent the host from converting LC3 labeling into effective killing.
- The parasite dynamically transitions its strategy across developmental time: early evasion of LC3-dependent restriction precedes late-stage exploitation of GABARAP–TFEB signaling.

This integrated framework redefines the liver stage as a competitive interface between noncanonical autophagy-mediated restriction and autophagy-driven metabolic exploitation, laying the foundation for the therapeutic approaches discussed in Section 4.

## 4. Therapeutic Modulation of Autophagy

Given the dual and highly specialized roles of autophagy during *Plasmodium* liver-stage infection—where CASM-driven PAAR restricts parasites while GABARAP–TFEB signaling supports parasite growth—the therapeutic potential of autophagy modulation depends on selectively enhancing host-protective pathways while avoiding reinforcement of parasite-beneficial mechanisms. This emerging paradigm underpins host-directed therapy (HDT), which aims to manipulate hepatocyte pathways that govern parasite fate while reducing the selective pressure imposed by parasite-targeted drugs.

### 4.1. Enhancing PAAR/CASM-Mediated Parasite Clearance

Recent studies demonstrate that liver-stage parasite elimination is mediated by noncanonical autophagy, reliant on the V-ATPase-ATG16L1-LC3 axis [16]. CASM activation begins at invasion, when membrane tension and ionic perturbation recruit the ATG16L1 WD40-domain to the PVM [15]. Agents capable of stabilizing LC3 lipidation or disrupting UIS3-LC3B interaction—a central parasite evasion mechanism [12]—represent promising HDT candidates. Strengthening PAAR execution without stimulating canonical autophagy may enhance parasite elimination while minimizing off-target metabolic effects.

### 4.2. Preventing Parasite Exploitation of Host Autophagy

While LC3-driven PAAR restricts parasites, liver-stage *Plasmodium* can exploit GABARAP paralogs to activate TFEB, the master regulator of lysosomal biogenesis [20]. This creates a nutrient-rich intracellular environment favorable for growth. Thus, autophagy activators that globally elevate ATG8-family activity could inadvertently enhance parasite survival. Selective inhibitors that disrupt GABARAP–TFEB coupling while preserving LC3-dependent PAAR represent a more precise HDT strategy.

### 4.3. Repurposing Existing Autophagy Modulators

Several autophagy-modulating drugs offer repurposing potential:

- Rapamycin enhances autophagy through mTOR inhibition and reduces the liver-stage burden, although immunosuppressive effects limit its translational viability [33].
- Chloroquine (CQ)/hydroxychloroquine (HCQ) inhibit lysosomal acidification and may disrupt parasite access to nutrients, but resistance and toxicity constrain their use [9].
- Carbamazepine (CBZ) induces mTOR-independent autophagy, potentially enhancing hepatocyte resilience [34].
- Metformin activates AMPK and reprograms hepatocyte metabolism, reducing liver-stage replication [18].

These observations demonstrate that manipulating autophagy yields context-dependent effects, underscoring the need for pathway-selective approaches.

### 4.4. Integrating Host Autophagy Modulators with Artemisinin-Based Therapies

Because artemisinin resistance is associated with enhanced stress tolerance and metabolic dormancy, combining ACTs with PAAR-enhancing therapies may accelerate parasite elimination and limit the emergence of resistant blood-stage clones [35]. Strengthening hepatocyte-driven clearance before parasites reach the bloodstream reduces parasite biomass and restricts the evolutionary space available for resistance selection [36].

### 4.5. Challenges and Prospects for Autophagy-Targeted Host-Directed Therapies

To complement the mechanistic and strategic framework presented above, Table 3 summarizes candidate host-directed therapeutics (HDTs) that target autophagy-related pathways in the liver stage. The table highlights both clinically available drugs and experimental molecules, together with their mechanistic basis and translational potential.

Challenges for autophagy-targeted therapies include hepatotoxicity risk, inter-individual variation in autophagy gene polymorphisms, and the difficulty of manipulating CASM without perturbing essential hepatocyte physiology. Advances in ATG8-selective chemistry, TFEB inhibitors, and humanized liver models may enable the development of precise autophagy modulators that enhance PAAR while preventing parasite exploitation.

Collectively, these insights position CASM-targeted HDT as a promising strategy with the potential to block liver-to-blood stage transition, reduce artemisinin resistance, and create evolution-resilient therapeutic combinations.

## 5. Knowledge Gaps and Future Perspectives

Despite major advances in understanding the interaction between *Plasmodium* liver-stage parasites and hepatocyte autophagy, several key gaps remain unresolved, limiting our ability to design targeted HDTs and to predict how different autophagy pathways influence infection outcome.

### 5.1. Unresolved Molecular Triggers of PAAR/CASM Activation

Although recent studies demonstrate that noncanonical autophagy is initiated during productive invasion via a V-ATPase-ATG16L1-dependent mechanism [15], the upstream sensing events that distinguish productive invasion from abortive entry or phagocytic uptake remain unclear. The precise membrane tension, ionic perturbations, and lipid compositions that activate CASM in hepatocytes have not been fully defined. Moreover, the influence of hepatocyte heterogeneity on PAAR initiation has not been examined. A deeper molecular and biophysical understanding of CASM activation at the PVM is needed to harness this pathway therapeutically.

### 5.2. Incomplete Understanding of Parasite Evasion Pathways

While UIS3-LC3 binding and UIS4-dependent actin remodeling are established as major evasion strategies [12,18], the extent to which these pathways act redundantly or synergistically remains unknown. It also remains unclear whether *Plasmodium* expresses additional PVM proteins that antagonize PAAR or prevent endolysosomal tethering. The structural basis of the UIS3-LC3 interaction is only partially characterized, and high-resolution structural studies could enable the rational design of UIS3 inhibitors. Furthermore, the dynamics of the perivacuolar actin network have never been visualized in vivo, representing a major gap in our understanding of evasion mechanisms.

### 5.3. Uncharacterized Functions of ATG8 Paralog Selectivity

Emerging evidence shows that *Plasmodium* can differentially recruit LC3 versus GABARAP paralogs to the PVM, enabling the parasite to block PAAR while simultaneously exploiting host TFEB-driven lysosomal expansion [20]. However, the determinants of this paralog selectivity are unknown. Whether ATG4 isoforms, lipid microdomains, or parasite-derived lipids shape ATG8 recruitment remains to be elucidated. Since ATG8 proteins have distinct roles in autophagy, membrane dynamics, and vesicular trafficking, understanding paralog-specific interactions is essential for developing selective HDTs.

### 5.4. Limited Translational Tools for Targeting CASM and PAAR

Although recent work shows that hepatocyte killing of liver-stage parasites depends on noncanonical autophagy [16], no selective CASM activators or ATG16L1 modulators are currently available. Most known autophagy drugs broadly target mTOR, AMPK, or lysosomes and therefore risk enhancing parasite exploitation pathways (e.g., the GABARAP-TFEB axis) [37,38]. Likewise, no pharmacological inhibitors of UIS3-LC3 interaction or UIS4-actin remodeling currently exist. Thus, there is an urgent need for chemical probes that selectively modulate LC3 lipidation, ATG16L1 localization, or TFEB activity in infected hepatocytes.

### 5.5. Modeling Barriers and Inter-Individual Variability

The lack of physiologically relevant models constrains progress in the field. Most mechanistic studies use transformed hepatoma lines, which differ substantially from primary hepatocytes in lipid metabolism, autophagy flux, and endolysosomal dynamics [39,40]. Humanized liver mouse models address some limitations but remain costly and low-throughput [41,42]. Although these mechanisms are robustly supported in *Plasmodium berghei* models, their conservation and quantitative contribution during *Plasmodium falciparum* liver-stage infection in humans remain incompletely defined.

Additionally, common human polymorphisms in autophagy genes—such as ATG16L1 T300A—may influence host susceptibility to liver-stage infection yet remain unexplored in malaria [35]. Understanding host genetic variability will be crucial for predicting patient-specific responses to HDTs.

### 5.6. Future Directions

Future research should prioritize dissecting CASM activation mechanisms, resolving the structural interactions between UIS3/UIS4, characterizing ATG8 paralog selectivity, and developing selective autophagy modulators compatible with HDT. Although this review focuses on *Plasmodium* liver stages, related apicomplexan parasites also interface with host autophagy and endolysosomal pathways. Comparative work across these intracellular vacuolar pathogens may help identify conserved versus *Plasmodium*-specific principles of autophagy engagement and immune evasion. Integrating single-cell transcriptomics, CRISPR perturbation screens, humanized liver platforms, and high-resolution imaging of the PVM will be key to generating a comprehensive map of hepatocyte defense pathways. Ultimately, defining the autophagy landscape of the infected hepatocyte—and how parasites rewire it—will accelerate the development of evolutionary-resilient interventions capable of blocking liver-stage progression and reducing the emergence of artemisinin resistance.

Together, these unresolved questions highlight that although substantial progress has been made in defining the molecular architecture of hepatocyte–*Plasmodium* interactions, our understanding of liver-stage malaria remains incomplete. The outstanding gaps—ranging from the upstream triggers of CASM activation to the structural basis of UIS3–LC3 sequestration to the determinants of ATG8 paralog selectivity and the limitations of current hepatocyte model systems—underscore the complexity of the autophagy landscape that shapes parasite fate. Addressing these knowledge gaps is not only critical for clarifying the fundamental biology of liver-stage infection but also essential for enabling the design of next-generation host-directed therapies that selectively reinforce protective autophagy while preventing parasite exploitation of those same pathways.

In light of these scientific and translational challenges, it becomes increasingly important to synthesize the emerging conceptual framework and articulate its implications for malaria biology and intervention. The following section integrates these mechanistic insights and outlines the broader significance of autophagy—both as a determinant of hepatocyte-intrinsic immunity and as a target of parasite manipulation—providing a unified perspective on how these processes can be leveraged for more effective malaria control strategies.

## 6. Conclusions

Recent advances have substantially refined our understanding of how autophagy influences *Plasmodium* liver-stage infection. Current evidence indicates that hepatocytes do not rely on canonical xenophagy; instead, they activate a noncanonical, CASM-dependent pathway that promotes LC3 lipidation on the parasitophorous vacuole membrane through a V-ATPase-ATG16L1 mechanism. This PAAR response represents a critical component of cell-intrinsic immunity and contributes to early parasite restriction.

At the same time, *Plasmodium* liver-stage parasites employ dedicated strategies to counteract this defense. UIS3-mediated sequestration of LC3 and UIS4-driven actin remodeling limit the execution of PAAR and maintain PVM integrity. Moreover, parasites can exploit host autophagy components—particularly GABARAP paralogs and TFEB-associated lysosomal remodeling—to enhance their access to nutrients and support intracellular development. These findings demonstrate that autophagy serves both protective and permissive functions, depending on the specific molecular interactions occurring at the host–parasite interface.

This duality underscores the necessity for therapeutic approaches that differentiate between beneficial and parasite-supportive autophagy pathways. Strategies that selectively reinforce CASM-mediated LC3 lipidation, while avoiding activation of GABARAP–TFEB signaling, may offer a targeted route for host-directed intervention. Such approaches have the potential to limit liver-to-blood stage progression and reduce selective pressures that facilitate antimalarial resistance.

Despite this progress, several challenges remain. Key unresolved questions include the upstream triggers of CASM activation, the breadth of parasite effectors contributing to PAAR evasion, and the determinants of ATG8-paralog specificity at the PVM. In addition, current hepatocyte models do not fully reproduce the metabolic and immunological complexity of human liver tissue. Advances in single-cell analysis, gene perturbation technologies, and humanized liver platforms will be instrumental for addressing these gaps.

Beyond their mechanistic implications, these insights have direct relevance for global malaria control efforts. A deeper understanding of hepatocyte-intrinsic immunity and parasite evasion pathways supports the development of innovative host-directed strategies aligned with the United Nations Sustainable Development Goal (SDG) 3—particularly Target 3.3, which aims to end malaria as a public health threat by 2030. By informing therapeutic approaches that reduce liver-stage progression and limit the emergence of drug resistance, research in this area contributes not only to basic science but also to long-term global health priorities.

## Figures and Tables

**Table 1:** Summary of host PAAR/CASM mechanisms against *Plasmodium* liver stages.

Mechanism/Component	Molecular Description	Functional Outcome for Host	Key Evidence	References
CASM activation via the V-ATPase-ATG16L1 axis	Noncanonical autophagy pathway in which ATG16L1 is recruited to the single-membrane PVM, promoting direct LC3 lipidation independent of ULK1-Beclin1-VPS34	Initiates the PAAR response; primes PVM for lysosomal fusion	Essential for early parasite restriction	[15,28]
LC3B lipidation on the PVM (PAAR initiation)	Persistent LC3B conjugation onto PVM surface; not associated with canonical autophagosome formation	Marks PVM as a target for degradative pathways; recruits endolysosomal machinery	Long-lasting LC3 decoration observed in infected hepatocytes	[9,14,27]
Endolysosomal fusion with LC3-decorated PVM	Lysosomes tether and fuse with the LC3-positive PVM	Parasite degradation; NO-independent killing	Confirms LC3-driven parasite elimination	[10,24]
Host ubiquitin and adaptor recruitment	Ubiquitin and autophagy adaptors localize to PVM following LC3 loading	Enhances membrane damage sensing; supports PAAR maturation	Observed in high-resolution imaging	[12,15]

**Table 2:** Parasite evasion and exploitation strategies at the PVM.

Parasite Strategy	Molecular Mechanism	Impact on Host Autophagy	Net Effect on Parasite Survival	References
UIS3-mediated LC3 sequestration	UIS3 binds LC3B and prevents its engagement with degradative adaptors	Blocks execution of PAAR despite LC3 recruitment	Prevents lysosomal fusion and early killing	[12]
UIS4-induced actin remodeling	UIS4 remodels perivacuolar actin to create a physical barrier around the PVM	Restricts access to lysosomes, adaptors, and ubiquitin	Provides structural shield against PAAR	[18,19]
Differential ATG8 recruitment (LC3 vs. GABARAP)	Selective enrichment of GABARAP rather than LC3 at later stages	Suppresses LC3-mediated restriction while enabling metabolic rewiring	Supports replication and organelle biogenesis	[20]
Parasite-driven TFEB activation	GABARAP-dependent signaling activates the TFEB transcriptional program	Increases lysosomal biogenesis and membrane trafficking	Expands nutrient availability and supports rapid growth	[20,21,23]
Aquaporin-3 and cholesterol manipulation	Parasite manipulates host lipid/water channels and cholesterol pools	Alters hepatocyte homeostasis in favor of parasite metabolism	Supports membrane expansion and schizogony	[7,19]

**Table 3:** Host-Directed Modulation of Autophagy During *Plasmodium* Liver-Stage Infection.

Intervention Target	Mechanistic Rationale	Expected Effect on Liver-Stage *Plasmodium*	Evidence Type	References
V-ATPase–ATG16L1 axis (CASM enhancement)	Strengthens LC3 lipidation on the PVM and accelerates lysosomal fusion.	Increases PAAR-mediated parasite restriction.	Conceptual/mechanistic	[15,24]
UIS3–LC3 interaction blockade	Disrupts UIS3 sequestration of LC3, restoring progression of PAAR.	Promotes lysosomal engagement and parasite clearance.	Small-molecule in vitro	[13]
GABARAP–TFEB signaling inhibition	Prevents parasite-driven activation of lysosomal biogenesis.	Reduces nutrient and membrane supply for parasite growth.	Conceptual/mechanistic	[20,23]
mTOR inhibition (Rapamycin)	Induces autophagy and modulates hepatocyte stress response.	Decreases liver-stage parasite burden but risks immunosuppression.	In vivo rodent	[33]
AMPK activation (Metformin)	Reprograms hepatocyte metabolism and enhances host resilience.	Reduces parasite replication during liver development.	In vivo/mechanistic	[18]
Lysosomal acidification blockade (CQ/HCQ)	Disrupts parasite nutrient access via altered lysosomal pH.	Mixed effect; may impair parasite survival but limited by toxicity.	In vitro/clinical history	[9]
mTOR-independent autophagy induction (Carbamazepine)	Enhances autophagic flux independently of classical signaling.	Potential hepatoprotective effects; requires further validation.	In vitro	[34]
TFEB inhibitors (experimental)	Suppresses excessive lysosomal biogenesis exploited by parasite.	Expected to limit intracellular growth and schizogony.	Conceptual	[20]

## Data Availability

This article is a narrative review, and no new data were generated or analyzed. Therefore, data sharing is not applicable.

## Abbreviations

The following abbreviations are used in this manuscript:

ACTs	Artemisinin-based Combination Therapies
AMPK	AMP-Activated Protein Kinase
ATG	Autophagy-Related Gene
ATG4	Autophagy-Related Protein 4
ATG5	Autophagy-Related Protein 5
ATG8	Autophagy-Related Protein 8
ATG12	Autophagy-Related Protein 12
ATG16L1	Autophagy-Related Protein 16 Like 1
CASM	Conjugation of ATG8 to single Membrane
CBZ	Carbamazepine
CQ	Chloroquine
GABARAP	Gaba Type A Receptor-Associated Protein
HCQ	Hydroxychloroquine
HDT	Host-directed Therapy
LC3/LC3B	Microtubule-Associated Protein 1A/1B Light Chain 3
mTOR	Mechanistic Target of Rapamycin
NO	Nitric Oxide
PAAR	*Plasmodium*-associated autophagy-related
PV	Parasitophorous vacuole
PVM	Parasitophorous Vacuole Membrane
SDGs	Sustainable Development Goals
TFEB	Transcription Factor EB
UIS3	Upregulated in Infective Sporozoites 3
UIS4	Upregulated in Infective Sporozoites 4
